# Quantifying Risk for Anxiety Disorders in Preschool Children: A Machine Learning Approach

**DOI:** 10.1371/journal.pone.0165524

**Published:** 2016-11-23

**Authors:** Kimberly L. H. Carpenter, Pablo Sprechmann, Robert Calderbank, Guillermo Sapiro, Helen L. Egger

**Affiliations:** 1 Department of Psychiatry and Behavioral Sciences, Duke University School of Medicine, Durham, North Carolina, United States of America; 2 Electrical and Computer Engineering, Biomedical Engineering, and Computer Science, Duke University, Durham, North Carolina, United States of America; Sichuan University West China Hospital, CHINA

## Abstract

Early childhood anxiety disorders are common, impairing, and predictive of anxiety and mood disorders later in childhood. Epidemiological studies over the last decade find that the prevalence of impairing anxiety disorders in preschool children ranges from 0.3% to 6.5%. Yet, less than 15% of young children with an impairing anxiety disorder receive a mental health evaluation or treatment. One possible reason for the low rate of care for anxious preschoolers is the lack of affordable, timely, reliable and valid tools for identifying young children with clinically significant anxiety. Diagnostic interviews assessing psychopathology in young children require intensive training, take hours to administer and code, and are not available for use outside of research settings. The Preschool Age Psychiatric Assessment (PAPA) is a reliable and valid structured diagnostic parent-report interview for assessing psychopathology, including anxiety disorders, in 2 to 5 year old children. In this paper, we apply machine-learning tools to already collected PAPA data from two large community studies to identify sub-sets of PAPA items that could be developed into an efficient, reliable, and valid screening tool to assess a young child’s risk for an anxiety disorder. Using machine learning, we were able to decrease by an order of magnitude the number of items needed to identify a child who is at risk for an anxiety disorder with an accuracy of over 96% for both generalized anxiety disorder (GAD) and separation anxiety disorder (SAD). Additionally, rather than considering GAD or SAD as discrete/binary entities, we present a continuous risk score representing the child’s risk of meeting criteria for GAD or SAD. Identification of a short question-set that assesses risk for an anxiety disorder could be a first step toward development and validation of a relatively short screening tool feasible for use in pediatric clinics and daycare/preschool settings.

## Introduction

Early childhood anxiety disorders are common, impairing, and predictive of anxiety and mood disorders later in childhood [1–8]. Epidemiological studies over the last decade find that the prevalence of impairing anxiety disorders in preschool children ranges from 0.3 to 6.5% [8–11]. Yet, less than 15% of young children with an impairing anxiety disorder receive a mental health evaluation or treatment [10]. The ability to quickly and reliably detect and intervene with anxiety disorders, while the child’s brain is still developing, could directly alter the child’s developmental trajectory [12, 13] and may put the child at decreased risk for psychiatric illnesses later in life. One possible reason for the low rate of care for anxious preschoolers is the lack of affordable, timely, reliable and valid tools for identifying young children with clinically significant anxiety.

The Preschool Age Psychiatric Assessment (PAPA) is a comprehensive parent-report diagnostic interview that assesses symptoms for a range of psychiatric symptoms and disorders in children ages 2–5, including anxiety symptoms and disorders [11]. In this paper, we focus on two common anxiety disorders: generalized anxiety disorder (GAD) and separation anxiety disorder (SAD). We selected GAD and SAD because of their relatively high prevalence in early childhood [11, 14–18], with community samples suggesting that 2.7% of preschool age children meet criteria for SAD and 4.7% of preschool age children meet criteria for GAD [11]. As outlined in the PAPA codebooks (available at: http://devepi.mc.duke.edu/codebooks.html), calculations of symptom scale scores and diagnoses for GAD and SAD require up to 41 and 54 individual PAPA items, respectively. In a full PAPA interview yes/no intensity questions have follow-up questions about examples and frequency, duration, and onset questions, some of which are not used in the diagnostic algorithms; thus the actual number of items for each disorder is much higher. Additionally, a final designation of GAD or SAD using the full PAPA interview requires the symptoms to be associated with significant impairment, requiring additional questions about the domains of impairment. Although social anxiety disorder (formerly called social phobia) is also commonly identified in young children [11, 14–18], only two PAPA items are required to determine whether a child meets criteria for social anxiety disorder and therefore the application of the ADTree algorithm, or any other data reduction and feature selection technique, to the social anxiety disorder construct is not appropriate.

There is an unmet need for screening tools for preschool anxiety that can be used in pediatric primary care settings or care-giving facilities, such as daycare centers and preschools, to assess risk and determine when a child should be referred for a specialty mental health evaluation. The PAPA is a diagnostic interview, not a screening tool. It requires significant training to administer and a mean of an hour and 40 minutes to administer the entire interview [11]. Early childhood screening tools, such as the 99 item CBCL 1.5–5 and the Strength and Difficulties Questionnaire (SDQ), assess broad anxiety domains, not risk for specific anxiety disorders [19, 20]. The 34-item Preschool Spence Anxiety Scale does cover the symptomatic domains for the most common anxiety disorders, including generalized anxiety disorder and separation anxiety disorder, yet is too long for use in primary care settings [21].

Over the last decade, our team has conducted large longitudinal studies of representative populations of preschoolers recruited from pediatric primary care. Trained interviewers have conducted over 1,800 PAPAs with parents, primarily in their homes or in the Duke Center for Developmental Epidemiology (CDE) lab. In this paper, we apply an *alternating decision trees algorithm* (ADtrees) [22] to these rich (big) data to test whether individual PAPA items, or clusters of items, can predict, with high reliability and low false positive rates, whether a child is likely to have GAD or SAD.

We elected to use the ADtree algorithms because the decision rules produced by ADtrees are compact and simple to interpret: they require evaluating individual symptoms and combining the results in a simple manner. Additionally, the classification results have been shown to be comparable to that of other state-of-the-art algorithms [22, 23]. That being said, we do not argue that this is the only machine learning system that could be used for this task. Indeed, other feature selection algorithms could achieve similar performance in this particular task. However, the combination of simple decision rules with high level of classification accuracy makes the use of ADTrees very appealing for the study of medical conditions. For instance, these algorithms have been used for early diagnosis of dengue fever [24], modeling disease trait information [25], heart-disease diagnostics [22], and for shortening tools used for the diagnosis of autism [23, 26, 27]. The use of this method across so many clinical dimensions underscores the power of this approach in identifying meaningful correlations among items from commonly used tools, which may help empower clinical decision-making and may streamline screening for any number of medical and psychiatric conditions.

This machine learning approach is not without its difficulties, as has recently been highlighted by Bone, et. al. [28]. In their paper, Bone et al. discuss several conceptual and methodological problems that arise when using machine learning techniques in the context of behavioral science. They outline a list of standard and essential methodological guidelines, such as using representative training data or accounting for class unbalances, which need to be considered when using machine learning tools. These practices are mature in the machine learning literature, and their use is critical for obtaining meaningful results that are not only valid for the available training data but can be generalized to the broad (unseen) population. We outline how we implemented each of these suggestions in the methodological section of this paper. Another point raised by Bone et al in [28] is that machine learning techniques applied in the absence of clinical domain expertise can lead to erroneous or misinformed conclusions. Our multidisciplinary mental health and engineering collaboration emerged from our agreement with these authors and our belief that domain expertise must be coupled with the engineering expertise to discover meaningful and useful new tools, as well as to provide the proper interpretation of the machine results.

Through this work, we have applied ADtree algorithms to identify a reduced subset of the full PAPA interview items that can reliably identify a significant proportion of children who met criteria based on the full interview for GAD and SAD. Identification of “high value” items that can reliably identify a significant proportion of children who meet criteria for GAD or SAD can then be used to create a shorter *clinical* risk assessment screen for these disorders. We explored the validity of the resulting risk classes using Cantwell’s criteria for establishing the validity of psychiatric disorders in children [29]. These criteria suggest that children with a diagnosis (or in a risk class, as in this paper) should share a number of factors known to be associated with psychopathology, including psychosocial factors, demographic factors, biological factors, genetic and environmental factors, natural history, and response to intervention (Reviewed in [29]).

With our approach, our goal is not to replace comprehensive clinical assessment, but rather to identify the PAPA item set with the best signal to enable us to identify children needing further mental health evaluations in an efficient and scalable manner. Furthermore, we aim to develop a *probability* risk score, rather than a binary assessment, that quantifies the confidence with which subsets of items predict a specific disorder (not to be confused with the *severity* of the disorder). This approach will enable us to provide both the parent and clinician with information about how confident they can be that a child does or does not have GAD or SAD and whether or not they need further assessment and possible intervention. Furthermore, in the future, this approach may be used to quantify treatment response over time.

## Methods

### Study Designs and Participants

Data were acquired from two independent studies: (i) The Preschool Age Psychiatric Assessment (PAPA) Test-Retest Study (PTRTS) (n = 307, weighted to a screening population of 1,073 children) [2, 11] and (ii) The Duke Preschool Anxiety Study (PAS) (n = 917, weighted to a screening population of 3,433 children) [9]. Table 1 includes the demographic and associated characteristics of both of these samples. Both studies recruited parents with children ages 2 to 5 years old who attended Duke University Pediatric Primary Care Clinics for both well-child and sick-child visits. These clinics care for a diverse population of families, drawn from the city of Durham and the surrounding rural areas of Durham County. Inclusion criteria for both studies were (i) the child was between 24 and 71 months old, and (ii) the child attended the pediatric clinic during a screening period. Exclusion criteria were (i) the child was not accompanied by a parent/legal guardian who could provide consent, (ii) the parent/legal guardian lacked adequate fluency in English to complete a screener, (iii) the index child was known to have IQ < 70, autism, or other pervasive developmental disorders, (iv) the child’s sibling was participating in the study, and/or (v) the provider decided that the child was too medically ill at the visit for the parent to be approached about the study. The Duke University Medical Center Institutional Review Board has continuously approved both of these studies. Written informed consent from the parent was obtained following a complete description of the study.

**Table 1:** Demographic Distributions and Associated Characteristics of Study Participants.

		Training Sample: PAS		Testing Sample: PTRTS	
		Unweighted N (Overall N = 917)	Weighted Percent	Unweighted N (Overall N = 307)	Weighted Percent
**Age (years)**	2	276	27%	94	35%
	3	241	26%	73	22%
	4	172	18%	74	24%
	5+	228	29%	66	18%
**Sex**	Female	451	52%	141	48%
	Male	466	48%	166	52%
**Race**	African American	384	32%	168	59%
	Non-African American	533	68%	139	41%
**N of biological parents in home**	0	50	4%	24	10%
	1	326	25%	131	34%
	2	541	71%	152	56%
**N of siblings in home**	0	281	28%	82	19%
	1	395	45%	128	46%
	2	162	19%	60	24%
	≥3	79	7%	37	12%
**Poverty**	Below the federal poverty level	168^a^	12%	100^b^	28%
**Diagnoses**	Generalized Anxiety	175	9%	33	5%
	Separation Anxiety	194	11%	32	5%
	Social Anxiety	135	8%	23	4%
	ADHD	86	4%	22	3%
	Conduct Disorder	44	3%	24	5%
	ODD	106	6%	34	6%
	Depression	50	2%	17	3%
**Family History**	Psychiatric Diagnoses	445	40%	102	28%
	Substance Abuse	188	13%	72	14%
		**Unweighted Avg (Std)**	**Weighted Avg (Std)**	**Unweighted Avg (Std)**	**Weighted Avg (Std)**
**Impairment**	Number of impairments	1.6 (1.9)	1.0 (1.5)	1.2 (2.1)	0.7 (1.6)
**Episodes of Irritability in Last 3 Months**	Temper Tantrums	76 (188)	53 (137)	80 (144)	61 (130)
	Irritable Mood	116 (313)	71 (226)	143.4 (321)	83 (237)
**N of Stressors**	Type A events^c^	1.8 (1.0)	1.7 (0.9)	1.8 (1.0)	1.7 (0.8)
	Type B events^d^	0.6 (0.9)	0.5 (0.8)	1.0 (1.2)	0.9 (1.1)
**N of Associated Symptoms**	Sensory Sensitivity	0.5 (0.8)	0.3 (0.7)	0.2 (0.6)	0.2 (0.5)
	Sleep disturbances	4.5 (2.1)	3.8 (1.9)	3.9 (2.1)	3.4 (1.9)
	Physical symptoms	1.3 (1.4)	1.0 (1.3)	0.9 (1.1)	0.6 (1.0)

Key: PAS = Preschool Anxiety Study; PTRTS = PAPA Test-Retest Study; ODD = Oppositional Defiant Disorder,

^a^Missing information from N = 73.

^b^Missing information from N = 19,

^c^Includes new children in the home, parental divorce, moving, etc.,

^d^Includes major accidents and injuries, death of a loved one, sexual or physical abuse, etc.

#### The Preschool Age Psychiatric Assessment (PAPA)

The Preschool Age Psychiatric Assessment (PAPA) is a parent-report instrument for the assessment of psychopathology in 2-5-year-olds. The PAPA uses a highly structured protocol, with required questions and probes, however, the onus throughout is on the interviewer to ensure that the parent (1) understands the question being asked; (2) provides clear information on behavior or feelings relevant to the symptom; and (3) reports the symptom at a pre-specified level of severity as defined in an extensive glossary. When symptoms are reported, their frequency, duration, and dates of onset are collected to determine whether they meet the symptom and duration criteria for the various DSM diagnoses. A three month “primary period” is used, because shorter recall periods are associated with more accurate recall [30]. A trained interviewer completes the PAPA with a parent and then codes the PAPA items after the interview is completed. Computer algorithms written in SAS are run on the raw PAPA data to generate symptoms, symptoms scales, and diagnoses. The PAPA includes assessment of most DSM-IV diagnostic criteria insofar as they are relevant to younger children, plus all items in the Diagnostic Classification: 0-3R [31]. DSM-IV diagnoses include: attention-deficit/hyperactivity disorder, oppositional defiant disorder, conduct disorder, depression (major depression, dysthymia and depression-not otherwise specified), anxiety disorders (separation anxiety disorder, generalized anxiety disorder, social phobia, specific phobia, posttraumatic stress disorder, and selective mutism), and elimination disorder (enuresis and encopresis). The assessment of impairment resulting from each group of symptoms was based upon the World Health Organization’s International Classification of Functioning, Disability and Health [32]. The test-retest reliabilities of the PAPA is comparable to psychiatric interviews for older children, adolescents and adults [10, 11].

#### Training Sample—The Preschool Anxiety Study (PAS)

In PAS, 3,433 parents were screened using a 10-item anxiety screening measure developed using data from our earlier Test-Retest study [11]. A child was identified as screen positive if the parent endorsed 4 or more of the 10 items on the screening questions. All children who screened high (n = 944, 27% of total sample) and a random sample of 189 children who did not screen high were selected to participate in an in-home assessment. Of these 1,132 children, 917 parents (81%) completed the PAPA and checklist assessments about the child’s psychiatric symptoms and temperament, the parent’s own personality traits, symptoms of anxiety, and depression, and distress in the parent-child relationship. Subjects were assigned a weight inversely proportional to their probability of selection allowing us to generalize our results back to the large screening sample of 3,433 preschoolers. Interviewers were blind to the child’s screen status. Of the 917 children for whom a PAPA was completed, 15% of the weighted sample (unweighted n = 281) met symptom level and impairment criteria for either GAD or SAD. Additional information about the study design can be found in [9].

#### Testing Sample—The PAPA Test-Retest Study (PTRTS)

In the PTRTS, 1,073 parents with children ages 24 to 71 months completed the CBCL1/2-5 questionnaire [19]. Children who obtained a T-score greater than or equal to 55 on the total symptom score of the CBCL1/2-5, which identifies the top 30% of the general population according to the CBCL1/2–5 norms [19], were considered screen high. Stratifying by age, sex, and race, parents of 193 screen highs (18% of the screened sample) and a random sample of 114 screen lows were recruited and completed the interview phase (total n = 307). As in the PAS study, subjects were assigned a weight inversely proportional to their probability of selection and interviewers were blind to the parent’s screen status and to the results from the first interview at the time of the second interview. Our weighted data represents the screened population of 1,073 preschoolers. The mean interval between interviews was 11 days. Of the 307 children for whom a PAPA was completed, 7% of the weighted sample (unweighted n = 50) met symptom level and impairment criteria for either GAD or SAD. Additional information about the study design can be found in [11]. The first PAPA interview was used as the testing sample for the current study.

### Alternating Decision Trees

Alternating decision trees are an extension of both binary decision trees and voted stumps [22]. The structure of the ADtrees classifier is given by a series of decision nodes and prediction layers organized in the form of a tree. The decision nodes are given by very simple binary rules based on individual variables. They take the form of a yes/no question evaluating a symptom. A follow-up question might specify the frequency or duration of this symptom. Each decision node is followed by a prediction layer that assigns a real-valued coefficient to the each of the binary outputs of the decision rule. The classification of given instance is performed by following all paths for which all decision nodes are true and computing the sign of the sum of the coefficients of all the prediction layers that are crossed [22]. We will refer to the output of the algorithm as the *AD-score*. Choosing the class associated to positive (or negative) AD-score is arbitrary. In all of our experiments, we use a positive sign for the presence of a disorder and a negative sign for its absence. ADtrees use ADBoost [33] to learn the parameters on each individual classifier. The algorithm iteratively grows the tree by adding a decision node and a prediction layer one at a time; see [22, 33] for details. The total number of nodes in the tree is the only free parameter in the model and needs to be set using a model validation technique, as described in the next section.

### Selecting optimal parameters

The only parameter that needs to be tuned is the number of nodes in the tree, which corresponds to the number of items in the reduced questionnaire being learned. We performed cross-validation within the training data and selected the number of nodes by monitoring the performance on the left-out data at each round. This is standard practice in machine learning as adapting a classifier to the training set too closely can lead to over fitting, degrading the generalization ability of the model. In order to select the number of items in the reduced questionnaire, we ran 50 rounds of 10-fold cross-validation for both GAD and SAD, following the standard practice in the data mining community, for trees sizes varying between 2 to 15 nodes. This means that, at each fold, a random subsample containing 90% of the training data was used for fitting the model and the remaining part was used as validation. The final model was obtained using the whole available training set and using the number of nodes found with the cross-validation.

We used the PAS sample as our training set and then tested the algorithms on the independent PTRTS dataset: a completely different dataset with different subjects and different interviewers, but collected in an identical fashion from the same Duke Pediatric Primary Care clinics. This is clearly stronger than common leave-one-out type of validation techniques and very important for cross-site use of our results.

### Training setting

At the general population level, the presence and absence of anxiety disorders are two very unbalanced classes since only a combined 13% (weighted) of preschoolers meet criteria for GAD and/or SAD in our two previous studies [9, 11]. Consequently, training a classifier using samples from the general population would lead to unsatisfactory results. For example, classifying every child as not having an anxiety disorder would lead to a small misclassification rate, but all errors would be false negatives. In this design, the group of children who screened as at high-risk for anxiety was significantly over-represented since the children who screened as low risk were under-sampled.

### Risk score

As it was mentioned above, at testing, an instance is classified according to the sign of the AD-score. Intuitively, the absolute value of AD-score gives a measure of *confidence* in the classification results. This observation has been analyzed experimentally [34] and theoretically [35]. Rather than producing a hard binary decision, one can translate the AD-score into a risk score using the equation:

Risk Score = 1 – (1/(exp(AD−Score) + 1))

High-risk scores (close to one) reflect high confidence in the presence of the disorder, while low risk scores (close to zero) reflect high confidence in the absence of the disorder. The uncertain cases are associated with risk scores close to 0.5.

### Data Analysis

We measured performance of our ADTrees in terms of sensitivity, specificity, and accuracy (Acc) defined as the percentage of correct classifications:

Acc = (True Positives + True Negatives)/(False Positives + False Negatives + True Positives + True Negatives).

We defined a positive case, as identified using the ADTrees, as any case with a risk score greater than 0.5.

Using a framework to characterized the validity of pediatric psychiatric syndromes (Reviewed in [29]), we investigated the relationship between our risk probabilities, as derived from the machine learning trees, and a number of associated factors. These associated factors included: 1) The child’s level of impairment; 2) Comorbid disorders; 3) Other associated symptoms, such as irritability, sensory sensitivities, sleep problems, and physical symptoms like tummy aches; 3) Family risk factors, namely family history of psychiatric diagnoses or substance abuse; 4) Number of lifetime stressors, including type A stressors (e.g. new children in the home, parental divorce, moving, etc.) and type B stressors (e.g. major accidents and injuries, death of a loved one, sexual or physical abuse, etc.); and 5) Socioeconomic risk factors known to co-occur with childhood anxiety disorder.

To avoid any bias, we elected to focus this secondary analysis on the results of our testing sample only, as opposed to exploring these relationships in both our testing and our training samples. Because the data were non-normally distributed, risk scores were converted to a gamma distribution with the equation *risk score/(1-risk score)*, and the relationship between risk scores and the associated factors were tested with the general linear model with a log linking function as implemented in the SAS genmod process. All statistics from this analysis are reported as Type 3 likelihood ratios.

### Baseline comparisons

While the main focus of this work is not to show that the ADTree approach is the best possible machine learning algorithm, it is still important to provide performance comparisons against a strong baseline to put the results into context. With this in mind, we compared the results obtained with ADTree with those produced by a state-of-the-art machine learning algorithm. In [23], the authors compared 15 different machine learning algorithm in the context of the diagnosis of autism, showing that ADTrees is a highly competitive alternative. In the current work, we compare the obtained results with one of the strongest alternatives evaluated in [23], namely the J48 algorithm. The J48 algorithm is an implementation of C4.5, which is a very strong baseline for classification tasks. C4.5 learns a decision tree (as in ADTree) where splitting criterion is the normalized information gain (difference in the entropy). This algorithm does not explicitly favor solutions with smaller number of questions. We enhanced this baseline by applying a pre-processing in which a subset of the questions are selected considering their individual predictive ability along with the degree of redundancy between them. Specifically, we used the CBF subset evaluation criterion combined with greedy stepwise search. We performed the same cross-validation strategy as detailed for the ADTree in order to obtain the best set of hyper-parameters.

## Results

### Final ADtrees for GAD and SAD

**Fig 1:**
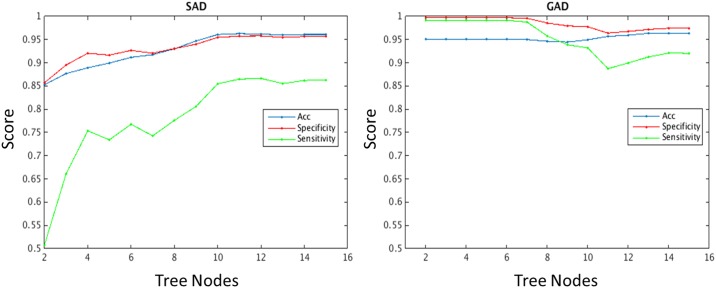
Average accuracy, specificity, and sensitivity by the number of nodes in each tree. During the training procedure, the number of nodes in the trees was chosen using 10-fold cross-validation. This figure shows the average accuracy, specificity, and sensitivity against the number of nodes in the tree for (left) SAD and (right) GAD. We chose to use trees with the smaller number of nodes producing the highest accuracy: 5 nodes for GAD, 10 nodes for SAD.

**Fig 2:**
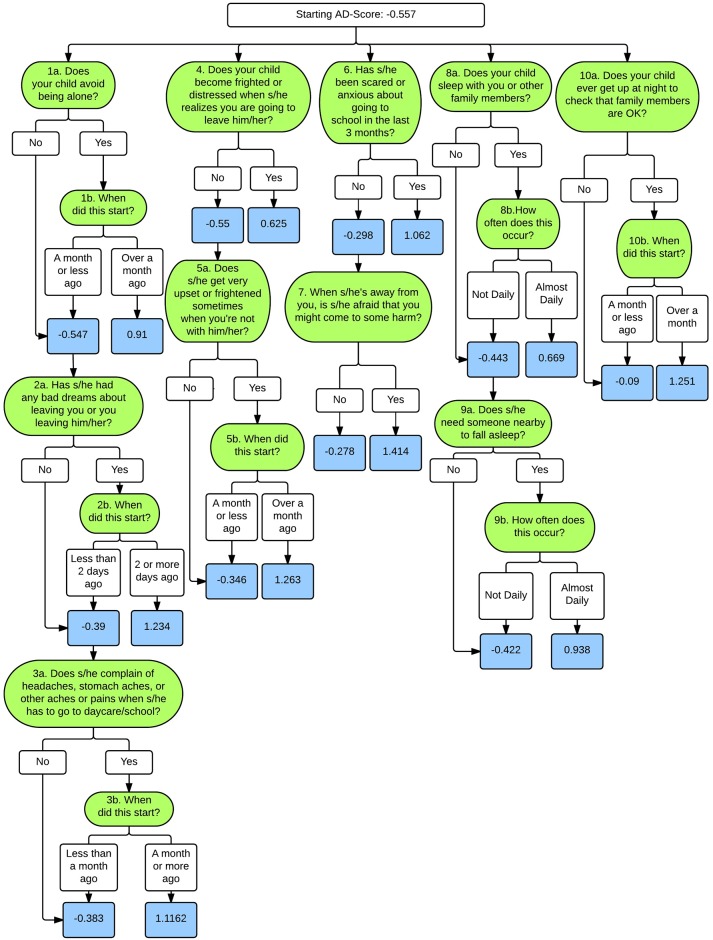
ADTree for Separation Anxiety Disorder. Green boxes represent individual PAPA items; white boxes represent decision points, and blue boxes represent the associated AD-Score.

Fig 1 shows the average sensitivity, specificity, and accuracy versus the number of nodes in each tree. For the SAD tree, all three quantities stop improving (and remain stable) after 10 nodes. As depicted in Fig 2, these 10 nodes translate into a minimum of 8 individual PAPA items and a maximum of 17 PAPA items.

**Fig 3:**
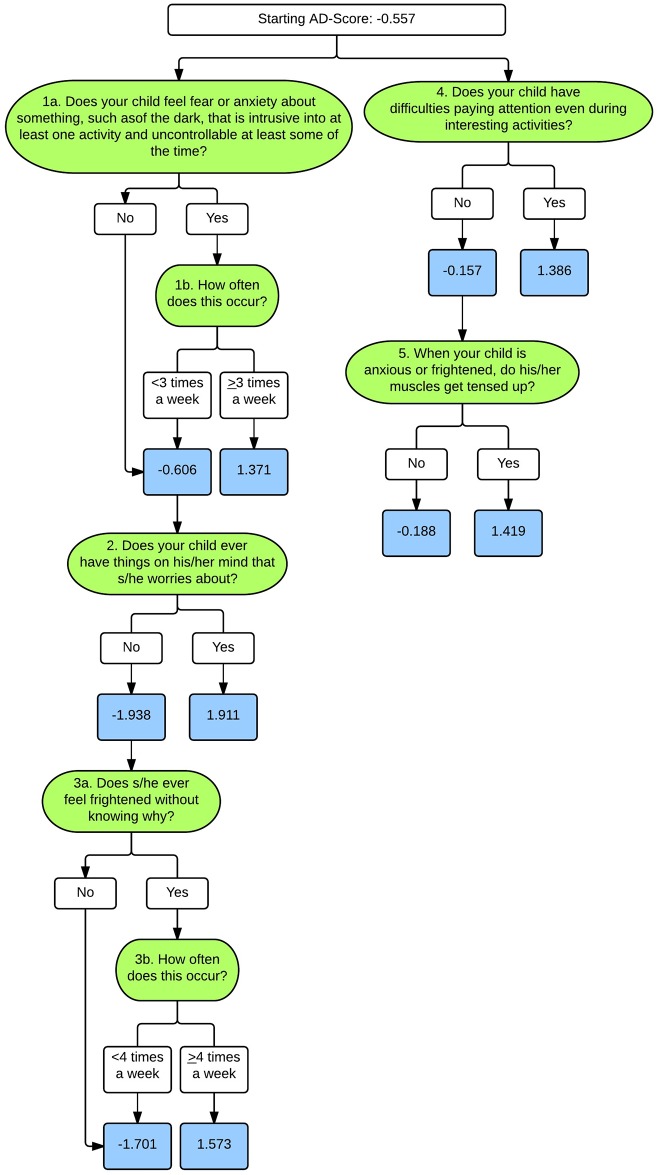
ADTree for Generalized Anxiety Disorder. Green boxes represent individual PAPA items, white boxes represent decision points, and blue boxes represent the associated risk AD-Score.

For the GAD tree, the accuracy is more or less constant for trees containing fewer than 5 nodes (Fig 1). Trees using more than 5 nodes achieve a slight improvement in accuracy at the expense of a reduction in both specificity and sensitivity. We prefer to have higher sensitivity (more conservative towards false negatives), given that the proposed method is not meant to substitute for a comprehensive psychiatric assessment. One could think of having a tree with only 2 nodes, given that the performance does not significantly improve. However, trees with too few items can lead to trees with less confidence (i.e. wider confidence intervals) around risk probabilities. Because of this, we chose to use 5 nodes for the GAD model. As depicted in Fig 3, these 5 nodes translate into a minimum of 3 individual PAPA items and a maximum of 7 PAPA items.

**Fig 4:**
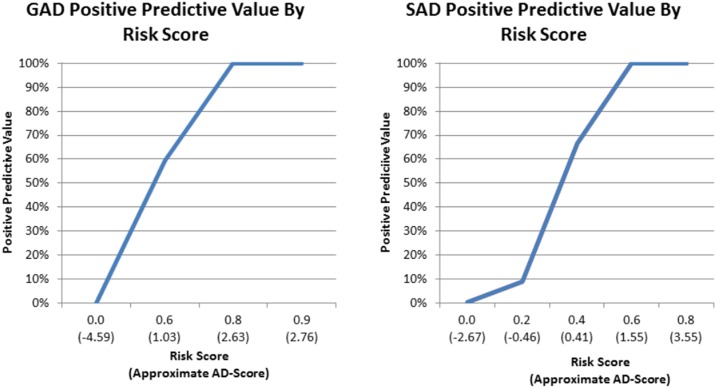
Positive Predictive Value by Risk Scores. Lines represent the weighted positive predictive value, which is the probability that the child meets criteria for Generalized Anxiety Disorder (GAD) and Separation Anxiety Disorder (SAD) on the full PAPA interview in the PTRTS test sample, for each Risk Score and associated AD-Scores.

**Fig 5:**
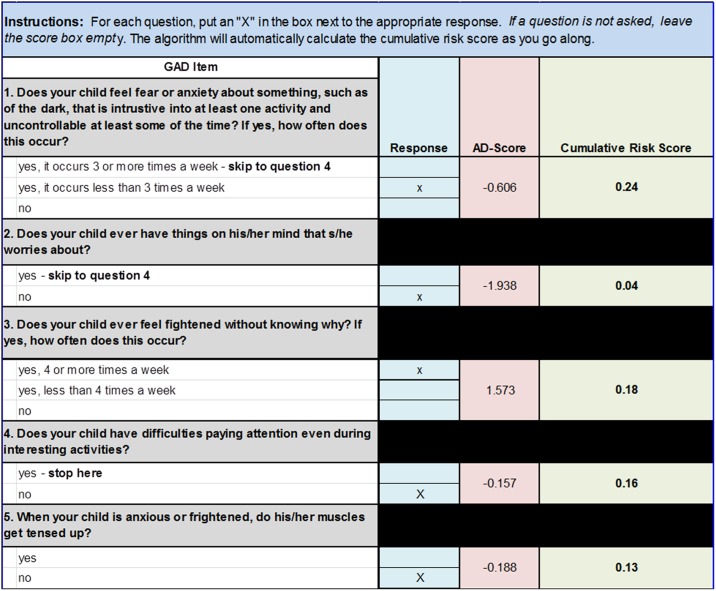
Example Clinical Screening Form. Example of an auto-scoring form populated with questions and AD-Scores from the GAD ADTree depicted in Fig 3. As the examiner answers the questions (denoted as an “X” next to the associated answer in the “Response” column), the score sheet automatically assigns the associated AD-Score and calculates a cumulative risk score using the equation: *Risk Score* = 1 − (1/(*exp*(*AD* − *Score*) + 1)) *with each additional question*.

Fig 4 shows the relationship between the risk score and the probability that the child met criteria for GAD (Fig 4A) or SAD (Fig 4B) on the full PAPA interview in the PTRTS test sample. As the risk score increases towards 1, the probability (weighted to the screening sample) that the child will meet criteria for the disorder approaches 100%. Using this risk score, the information included in the ADTrees, and the associated AD-scores, can be translated into a clinically instructive form. Fig 5 illustrates for the GAD ADTree how this approach can be translated into a clinical form that automatically calculates the cumulative risk for the GAD as one proceeds through the hierarchy of the ADTree items. This type of clinical tool will need to be tested for reliability, validity, and acceptability in a future clinical study. An equivalent form could be made from the SAD ADTree.

### ADTree Success and Validity in an Independent Cohort

Tables 2 and 3 summarize the predictive success using the diagnostic tree questions for GAD and SAD in the PTRTS dataset, our testing set. The algorithm provides a very reliable risk estimator with accuracy values of 97% and 99% (weighted to the screening sample) for GAD and SAD, respectively. Furthermore, in our testing data weighted back to the screening population, we had a sensitivity of 100% and a specificity of 97.2% for GAD and a sensitivity of 84.7% and a specificity of 99.8% for SAD.

**Table 2:** Classification results of the obtained models in an independent sample of children from the Duke PAPA Test-Retest Study (PTRTS) recruited in an identical fashion as the training sample.

	Generalized Anxiety		Separation Anxiety	
	High Risk	Low Risk	High Risk	Low Risk
Diagnosed	46	0	40	9
Not Diagnosed	7	254	1	257

**Table 3:** Predictive success of the obtained models in an independent sample of children from the Duke PAPA Test-Retest Study (PTRTS) recruited in an identical fashion as the training sample.

	Generalized Anxiety	Separation Anxiety
	Weighted Values	Weighted Values
Accuracy	97.4%	99%
Sensitivity	100%	84.7%
Specificity	97.2%	99.8%
Positive Predictive Value	74.9%	98.0%
Negative Predictive Value	100%	98.6%

### Comparison between ADTrees and J48 Algorithm

The sensitivity and specificity results obtained by the J48 algorithm for the PTRTS dataset are shown in Table 4. We observed that for GAD the performance matches that of the ADTree while in the case of SAD, J48 produces slightly worse results. Namely, weighted back to the screening population, J48 had a sensitivity of 73.0% and a specificity of 97.1% for SAD, lower than the 84.7% and 99.8% obtained using ADTrees, respectively. Furthermore, the decision trees obtained with J48 were significantly larger than those produced by ADTrees. As an example, the trees produced by J48 for SAD contained 42 nodes in contrast with the 10 nodes used by ADTree, rendering the obtained decision rules less interpretable. These observations go in line with results reported in the literature [22, 23]. As a final note, the questions selected by the J48 algorithm were highly correlated with those selected by ADTree. In particular, the biggest differences were observed for the SAD tree. Both algorithms selected a total of eight questions (for j48 the answers to these questions are combined several times through the 42-node decision tree), sharing five of them.

**Table 4:** Predictive success of J48 model in an independent sample of children from the Duke PAPA Test-Retest Study (PTRTS) recruited in an identical fashion as the training sample.

	Generalized Anxiety	Separation Anxiety
	Weighted Values	Weighted Values
Accuracy	97.4%	97.1%
Sensitivity	100%	73.0%
Specificity	97.2%	99.4%
Positive Predictive Value	74.9%	91.9%
Negative Predictive Value	100%	97.5%

### Associated Features

Table 5 presents the relationships between our SAD and GAD risk probabilities and factors known to be associated with anxiety disorders in children. We found that increased risk scores for GAD were associated with the higher rates of other comorbid disorders, including SAD (χ^2^(1, N = 307) = 3.84, p = 0.05), conduct disorder (χ^2^(1, N = 307) = 6.19, p = 0.01), and oppositional defiant disorder (χ^2^(1, N = 307) = 8.69, p<0.01). Increased risk scores for GAD were also associated with higher levels of impairment (χ^2^(1, N = 307) = 13.70, p<0.001), increased number of lifetime stressors (χ^2^(1, N = 307) = 3.93, p<0.05), increased sleep disturbances (χ^2^(1, N = 307) = 12.76, p<0.001), and increased physical symptoms (χ^2^(1, N = 307) = 10.38, p = 0.001).

**Table 5:** Relationship between risk probability scores for GAD and Sad and the associated characteristics in the independent PTRTS testing sample.

		Type 3 Likelihood Ratios	
		GAD	SAD
**Demographics**	Age	17.49**	10.53*
	Sex (F = Female, M = Male)	1.18	0.61
	Race (AA = African American; O = Not African American)	3.40	0.51
	Family income blow the federal poverty level	0.02	0.05
	Number of biological parents in the home	1.46	2.88
	Number of siblings in the home	4.13	1.80
**Family History**	Psychiatric Diagnoses	2.36	8.11**
	Substance Abuse	0.08	0.59
**Other Diagnoses**	Generalized Anxiety	-	33.76^†^
	Separation Anxiety	3.84*	-
	Social Anxiety	1.20	70.20^†^
	ADHD	1.96	0.39
	Conduct Disorder	6.19**	16.42^†^
	ODD	8.69**	23.86^†^
	Depression	2.17	7.52**
**Impairment**	Level of impairment	13.70^†^	40.38^†^
**Irritability**	Episodes of behavioral reactivity in last 3 months	0.57	29.98^†^
	Episodes of irritable mood in last 3 months	0.11	41.66^†^
**Lifetime Stress**	Type A life events	3.93*	54.52^†^
	Type B life events	1.88	21.08^†^
**Associated Symptoms**	Sensory hypersensitivities	2.79	42.06^†^
	Sleep disturbances	12.76^†^	208.26^†^
	Physical symptoms	10.38**	76.83^†^

Note: The Type 3 Likelihood Ratio represents the strength of association between each variable and the risk scores as computed by the ADTree algorithms. Higher likelihood ratios represented stronger associations. Key: GAD = Generalized Anxiety Disorder; SAD = Separation Anxiety Disorder; ODD = Oppositional Defiant Disorder,

*p≤0.05;

**p≤0.01;

^†^p≤0.001.

Similarly, increased risk scores for SAD were associated with the higher rates of comorbid disorders, including GAD (χ^2^(1, N = 307) = 33.76, p<0.001), social anxiety disorder (χ^2^(1, N = 307) = 70.20, p<0.001), conduct disorder (χ^2^(1, N = 307) = 16.42, p<0.001), oppositional defiant disorder (χ^2^(1, N = 307) = 23.86, p<0.001), and depression (χ^2^(1, N = 307) = 7.52, p<0.01). Increased risk scores for SAD were also associated with higher levels of impairment (χ^2^(1, N = 307) = 40.38, p<0.001) and increased family history of psychiatric disorders (χ^2^(1, N = 307) = 8.11, p<0.01). Furthermore, increased risk scores for SAD were associated with increased sensory hypersensitivities (χ^2^(1, N = 307) = 42.06, p<0.001), sleep disturbances (χ^2^(1, N = 307) = 208.26, p<0.001), physical symptoms (χ^2^(1, N = 307) = 76.83, p<0.001), and irritability (irritable mood: (χ^2^(1, N = 307) = 41.66, p<0.001), behavioral reactivity: (χ^2^(1, N = 307) = 29.98, p<0.001)). Finally, increased risk scores for SAD were associated with increased number of type A lifetime stressors (e.g. new children in the home, parental divorce, moving, etc.; χ^2^(1, N = 307) = 54.52, p<0.001) and type B lifetime stressors (e.g. major accidents and injuries, death of a loved one, sexual or physical abuse, etc.; (χ^2^(1, N = 307) = 21.08, p<0.001)).

## Discussion

Although anxiety disorders begin early in life, are associated with significant impairment [4, 8] and predict psychopathology and impairment later in life [1–8], there are few tools for identifying young children with anxiety disorders. In this paper, we used machine-learning algorithms to test whether subsets of items derived from an intensive assessment using a full diagnostic interview can reliably assess risk for early childhood anxiety disorders. A strength of our results is that we present experimental validation of this approach by training the machine learning algorithms on a large dataset collected from a community sample of preschool children and testing the resulting item trees on an independent community sample of children who were recruited in an identical fashion. Our encouraging results suggest that machine learning derived screening trees could be used to create valuable tools for screening young children with impairing SAD and GAD.

While there are a number of tools that assess mental health in young children, young children with impairing GAD and SAD often go undiagnosed and untreated [36]. This is in part due to the lack of efficient and diagnostically specific tools for use in primary care settings where children receive their medical care. The work presented in the current paper is a first step towards developing a tool that will hopefully fill this unmet need. Specifically, we have developed risk assessment algorithms that will enable clinicians to quickly and reliably assess children for GAD and/or SAD and refer children for disorder specific follow-up diagnostic assessments and treatment.

In addition to decreasing the number of questions, our approach allows us to assign a screening risk score to each child, which lies on a continuous spectrum from low to high probability of meeting criteria for the disorder. In other words, the method provides a natural measure of confidence in the obtained results, which can inform and empower clinical decision-making. The validity of the risk score as a confidence measure is well understood and has been previously studied empirically [33] and theoretically [34]. As the examiner moves through the questions in the tree, s/he can automatically calculate the confidence around the diagnostic risk. It is possible that a child will reach a “tipping point” of confidence where no additional items need to be asked, decreasing the time burden associated with the assessment. The continuous nature of this score also allows the practitioner to understand the cost and benefit to setting particular cut points for referring individual children. For example, as depicted in Fig 4, setting a cut point of risk score = 0.5 on the SAD risk assessment will result in the practitioner sending children with approximately 65% risk of meeting criteria for the disorder for full mental health assessments. This may not be a viable option in low resource settings where there is a dearth of early childhood mental health experts to assess these children. Thus, in such settings a higher cut point, such as 0.6, which is associated with a positive predictive value of 100% may be more appropriate. Conversely, the cut point may be set lower in high resource settings where the goal is assess every single child with risk for the disorder.

It is important to point out that the risk score is not necessarily linked to the severity of the disorder. While some correlation is to be expected, as more severe cases tend to be easier to identify due a higher number of symptoms, the training procedure does not use this information at any stage. In fact, some false negative scores could be obtained on patients presenting severe anxiety disorders manifested by symptom profiles that are not well represented in the training dataset used for constructing the model. Conversely, some cases with low severity might present combinations of symptoms associated with a high risk score and consequently high confidence in what the correct diagnosis should be.

There are real concerns associated with the application of machine learning techniques to clinical data [28]. We have sought to address these concerns in our approach. We brought together a team with expertise in computer analytics and preschool anxiety disorders. We have collaborated in an iterative fashion; the analytics were never separated from the clinical expertise and judgement. Additionally, because our training data and testing data came from completely independent, but complimentary, samples, we are not concerned about model over-fitting, which occurs when the same training data study is used in the testing sets. Finally, use of community sample in pediatric primary care rather than a clinical sample in psychiatric clinics means that our results are applicable to the setting where they will be used. Thus, because both the training and the testing data are from representative community studies, rather than clinical studies, our data were not biased towards one group (e.g., cases) over another and were not biased towards the most severe cases.

The approach and results presented here reflect strengths of our group’s program of research in the developmental psychopathology of early onset anxiety disorders. First, we trained the algorithms with data from a large, representative community sample of children using a structured diagnostic interview (PAPA) and then tested the resulting trees on a completely independent sample of children recruited in an identical fashion. Second, we developed diagnostic specific ADTrees based on comprehensive diagnostic interviews about the child (the PAPA), not non-diagnostic checklist measures. Third, we examined the associations between the probabilities derived from our risk assessment algorithms and known associated diagnoses, features and risk factors and were able to demonstrate good internal and external validity.

Our study has a number of limitations. One limitation is that we tested the reliability of the proposed methodology in a dataset in which the items were asked as part of a full psychiatric interview. We will need to test in future studies how these items performed when asked separately from the diagnostic interview without the intensive training needed to conduct the PAPA. Both our training and testing samples are representative and include children who meet criteria other psychiatric disorders, not GAD and/or SAD alone. While this is a strength of our study since our sample reflects how psychiatric disorders present in reality, there is the possibility that our risk score are capturing an underlying general psychopathology factor inherent to multiple disorders that share common features [37]. To address these limitations, we will need to test our risk assessment screen in a large validation study, similar to our program of research on the PAPA [38].

There are also potential limitations inherent in the methods we used. First, data were acquired from two studies with total subject numbers of 1,073 and 3,433. While these might look like *small* data for common machine learning applications, it is a large sample for child mental health. Second, choosing the number of folds in cross-validation is a known problem when training machine learning algorithms. There is a natural compromise in this decision: choosing a large number of folds implies that the models trained on each fold use more training data (making the conclusions more likely to generalize to the full-data setting). However, it also implies that the data used on each fold have larger overlap (results are thus more correlated) and consequently might bias the conclusions. In practice, 10-fold cross validation, as was used in the current study, is considered a good balance for datasets with size similar to the one used in this project.

Third, the ADtree is trained to optimize a given objective function over the training data. The classic choice in the literature is to use the exponential loss, which is a convex approximation of misclassification rate [39]. This criterion can be poor when the class-distributions are highly unbalanced, such as is the case in the current dataset, as considering equal error costs implicitly emphasizes the majority class. There are several strategies to address this problem in the machine learning literature. One option is to select the parameters that maximize a robust classification performance metric, such as the unweighted average recall, as suggested in [28]. In this work, however, we mitigate this problem by adopting a data-sampling technique instead [40]. The idea is to use a sampled version of the training data in order to modify the class balance. A classifier trained in this way will perform a little bit worse on the most frequent class but considerably better in the infrequent one. An extreme example of this technique would be to train the model in a class-equalized setting, using the same number of samples for each class regardless of their true distribution.

By using the data-sampling technique employed in this study, the obtained sample is likely to be much better balanced, since children screening high are more likely to be diagnosed with an anxiety disorder than those screening low. On the other hand, sub-sampling the group of children who screened low allows the classifier to perform better on the more challenging cases. While screen low children comprise a very large part of the general population, the data is simpler (since most times none or very few symptoms are present) and can consequently be better represented with fewer examples.

Despite these limitations, there are several appealing properties of ADtrees that result in decision rules that are easy to interpret. First, the meaning of each decision node can be understood in isolation, as the AD-score is obtained by summing the contributions of the individual nodes. A node contributes a positive value to the AD-score when the condition that it evaluates is a predictor of the disorder under consideration. For instance, this is the case of node 1 of the SAD ADTree (Fig 2): if the child avoids being alone and this behavior has started more than 30 days before the date of the interview, the node contributes a positive value to the score and we can conclude that this symptom predicts SAD.

In a similar way, one can interpret the meaning of each decision node in the tree. After analyzing each decision node in isolation, one can further interpret the interactions between the different nodes in the tree. Nodes that appear in different branches of the trees present little to no interaction. For instance, from the GAD ADTree shown in Fig 3, we can observe that having difficulties paying attention during interesting activities (node 4) is an indicator of the disorder irrespectively of having things on the child’s mind that s/he worries about (node 2). In contrast, nodes placed within the same branch of the trees evaluate correlated symptoms. The significance of the nodes in the lower levels of the trees depends on the evaluation of their ancestral nodes. Looking back at the ADTree obtained for GAD, when the criterion of node 1 (fear or anxiety) is met, we have enough information to make the prediction and it is not necessary to evaluate node 2 (worries), conversely, only when the criterion of node 1 is not met is it worthwhile assessing the question given in node 2. While these interpretations are crucial to understand the method and asses its clinical relevance, it is important to highlight that the obtained decision rules should not be considered universal. The obtained decision tree is optimal for ADTree given the available training data, but it is not the only set of questions that could lead to accurate predictions.

In summary, we used machine learning technology to address the complex challenge of developing mental health screening tools for young children that are feasible for use in real-world settings. Our goal is to increase access to screening by decreasing the time it takes to assess risk and decreasing the amount of training required to administer the assessment. By identifying children early in the course of GAD and/or SAD, we hope that we can provide early access to treatments during this period of life when the brain is developing and emotion regulation capacities are being established. Increased access to screening and early identification must be linked with increased access to comprehensive evaluation and evidence-based interventions. Unfortunately, these resources are scarce even in high-resourced settings. By providing clinicians with a quantitative measure of risk probability, we can help them to make informed, patient-centered decisions about care for very young children within the context of resources available. Screening tools for preschool anxiety disorders will also raise awareness about the prevalence of anxiety disorders in young children and support advocacy for increased access to mental health evaluations and evidenced-based intervention for young children.

The opportunity to assess risk for impairing psychiatric disorders in young children not only has implications for individual children, but also for population level screening. Our hope is that, by further developing our screening algorithms into short risk assessment tools that can be implemented quickly and reliably, we will be able to collect data on much larger cohorts of children. This will enable us to better quantify risk for these disorders at the population level, understand their developmental, and advocate for increased services for the families impacted by these disorders.
